# NIR-II fluorescence in lung cancer: advancing precision diagnosis and image-guided therapy

**DOI:** 10.3389/fchem.2025.1720197

**Published:** 2026-01-14

**Authors:** YunLong Yang, Jiang Fu, RangPing Xie, Chun Wang, Sen Wang, Long Wen, Yang Yang, ChengKuan Liu, GuiYan Yi, Li Yu, HaiNing Zhou

**Affiliations:** 1 Department of Thoracic Surgery, Suining Central Hospital, Suining, Sichuan, China; 2 Medical Imaging Key Laboratory of Sichuan Province, North Sichuan Medical College, Nanchong, Sichuan, China; 3 Institute of Surgery, Graduate School, Chengdu University of Traditional Chinese Medicine, Chengdu, China; 4 Institute of Surgery, Graduate School, North Sichuan Medical College, Nanchong, China; 5 Department of Thoracic Surgery, Affiliated Hospital of Southwest Medical University, Luzhou, China; 6 College of Medical Technology, Chengdu University of Traditional Chinese Medicine, Chengdu, China; 7 Department of Physical Examination, Suining Central Hospital, Suining, Sichuan, China

**Keywords:** diagnose, integration, lung cancer, NIR-II, therapy

## Abstract

Lung cancer ranks among the leading causes of cancer incidence and mortality worldwide. Conventional diagnostic and therapeutic approaches remain constrained by suboptimal sensitivity, exposure to ionizing radiation, and poor overall prognosis, driving efforts to develop novel diagnostic and therapeutic strategies to improve clinical outcomes. Second near-infrared window (NIR-II) fluorescence imaging offers deep tissue penetration and high spatial resolution; accordingly, a wide array of NIR-II fluorescent probes, imaging systems, and photointerventional therapies have been developed. In this review, we summarize recent advances and applications of NIR-II in lung cancer diagnosis and treatment and discuss the design and future directions of integrated diagnostic-therapeutic (theranostic) platforms.

## 1 Introduction

Lung cancer remains among the most common malignant neoplasms worldwide. IARC GLOBOCAN 2022 estimated approximately 2.48 million new lung cancer cases (12.4% of all cancers) and 1.82 million deaths (18.7% of all cancer deaths) worldwide in 2022, placing lung cancer among the leading cancers in both incidence and mortality (Forde et al., 2022). Early detection is critical for improving outcomes. However, early-stage disease is frequently asymptomatic, lacks specific biomarkers, and is difficult to detect using conventional chest radiography and computed tomography; these imaging modalities also expose patients to ionizing radiation. Consequently, approximately 56% of patients present with distant metastases at diagnosis (Issanov et al., 2024). Despite notable advances in surgery, chemotherapy, targeted therapy, immunotherapy, and radiotherapy over the past decade, the 5-year overall survival rate for lung cancer remains approximately 19% (Li M. Y. et al., 2021), highlighting an urgent need for improved strategies for early detection and intervention.

**FIGURE 1:**
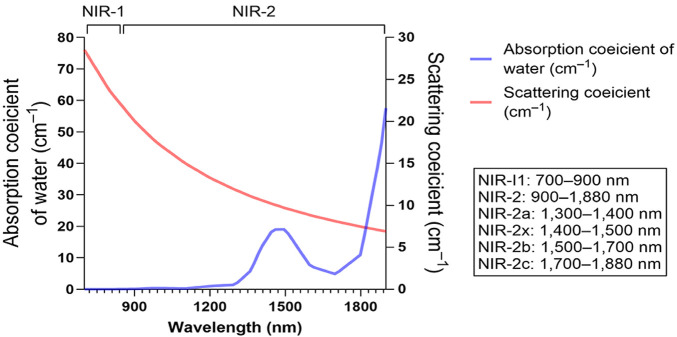
The principal bio-optical advantage of the NIR-II is substantially reduced light scattering in tissue relative to visible light and the NIR-I, which enhances spatial resolution and imaging depth. However, a pronounced water-absorption band centered near 1,400–1,500 nm can attenuate signal and degrade imaging performance within that sub-band.

Recent advances have established near-infrared (NIR) imaging as a valuable modality in precision oncology for lung cancer, owing to its deep tissue penetration, real-time guidance capability, potential for targeted therapy, and compatibility with nanotechnology-based approaches. The NIR-I window is conventionally defined as 700–900 nm. In microsurgery, indocyanine green (ICG)-enhanced NIR-I imaging is routinely used to assess vascular anastomotic patency and flap perfusion (Li X. et al., 2020; Wu et al., 2022; Zhang Z. et al., 2022). The NIR-II window typically spans approximately 900–1880 nm (some studies report 1000–1700 nm) and provides greater tissue penetration and an improved signal-to-noise ratio, facilitating enhanced tumor delineation in preclinical glioma models and enabling non-invasive brain imaging applications (Smith et al., 2009; Welsher et al., 2009; Li C. et al., 2020; Feng et al., 2021; Wang et al., 2022). NIR-II is further subdivided into NIR-IIa (1,300–1,400 nm), NIR-IIx (1,400–1,500 nm), NIR-IIb (1,500–1700 nm), and NIR-IIc (1700–1880 nm) (Diao et al., 2015; Feng et al., 2021; Hong et al., 2014) (Figure 1). Compared with NIR-I, NIR-II exhibits markedly reduced photon scattering and tissue absorption, as well as lower tissue autofluorescence, resulting in reduced image distortion, improved spatial resolution, and increased imaging depth (Zhao et al., 2020). Consequently, NIR-II fluorescence imaging is operationally straightforward, highly sensitive, free of ionizing radiation, and well suited for real-time *in vivo* monitoring to support clinical diagnosis and intraoperative guidance; it is also compatible with higher maximum permissible exposure (MPE) thresholds (Huang and Pu, 2020b; Shinn et al., 2021). The first-in-human NIR-II–guided tumor resection reported by Hu et al. demonstrated clinical feasibility (Hu et al., 2020). Moreover, NIR-II phototherapy—owing to reduced scattering and the ability to tolerate higher irradiation exposure—has demonstrated enhanced efficacy against solid tumors in preclinical studies (Dai et al., 2021b).

Deploying NIR-II for accurate lung cancer diagnosis and therapy represents a promising frontier. In this review, we emphasize recent diagnostic advances in NIR-II and emerging NIR-II–based therapeutic strategies that complement conventional treatments. We also discuss translational challenges, future prospects for clinical application, and the potential of integrated diagnostic–therapeutic platforms in improving lung cancer management.

## 2 Application of NIR-II in lung cancer diagnosis

### 2.1 High-sensitivity tumor imaging

Conventional radiologic modalities such as chest radiography and computed tomography (CT) have limited sensitivity and/or specificity for detecting early-stage lung cancer; CT also entails ionizing radiation and carries a risk of contrast-induced nephrotoxicity, and elevated false-positive rates can prompt unnecessary invasive procedures (Thanoon et al., 2023).

**FIGURE 2:**
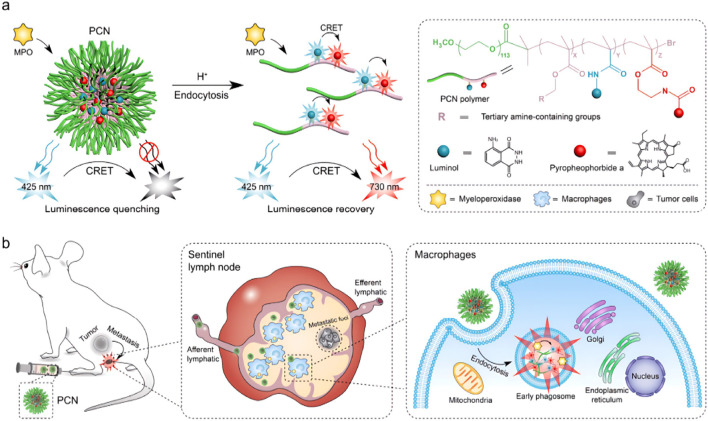
Schematic of a pH-amplified autoluminescent porous coordination network (PCN) incorporating a copolymer bearing a pH-responsive moiety and the fluorescent dye chlorin e6 (Ce6), enabling *in vivo* autoluminescence imaging for the detection of tumor metastases in sentinel lymph nodes. Copyright© 2021 Wiley-VCH GmbH. **(a,b)**.

NIR-II offers superior spatiotemporal resolution, higher signal-to-noise ratio (SNR), and increased tissue penetration owing to reduced tissue absorption, scattering, and autofluorescence (He et al., 2018; Huang and Pu, 2020a; Wan et al., 2019) (Table.1). NIR-II imaging can further differentiate tumors from adjacent normal tissues by sensing compositional and physiological contrasts—such as oxy-/deoxyhemoglobin, water, lipids, and blood oxygen saturation. Tumors frequently exhibit increased metabolic demand and aberrant perfusion, manifesting as altered hemoglobin distribution and hypoxia; these features are detectable in the NIR spectral region and can inform therapeutic selection and dose optimization (Choe et al., 2009; Kondepati et al., 2008; Yu et al., 2005; Zhou et al., 2007). Zhou et al. reported a cascade-activated, dual-quenching NIR-II probe (ACy-H-NTR) that sensitively detects tumor hypoxia and acidic microenvironments, thereby improving screening specificity and reducing false positives in lung cancer detection (Zhou et al., 2025). Elevated myeloperoxidase (MPO) activity in tumor-associated macrophages has also been exploited for early detection using luminol-mediated chemiluminescence imaging. Leveraging the acidic tumor microenvironment (TME), Wang et al. developed ultra-pH-responsive chemiluminescence resonance energy transfer (CRET) polymeric nanoparticles for noninvasive *in vivo* assessment of sentinel lymph node (SLN) metastasis. In this system, MPO-catalyzed luminol oxidation produces blue chemiluminescence; the luminol–pheophytin a (PPa) donor–acceptor pair transfers that energy via CRET to PPa, generating NIR emission and thereby enhancing the sensitivity and specificity of metastasis detection (Figure 2) (Wang et al., 2021).

**TABLE 1:** Comparison of NIR-I and NIR-II in terms of tissue penetration depth, signal-to-background ratio, and spatial resolution.

Classification	Tissue penetration depth	SBR	Spatial resolution (<1 mm)
NIR-I	1–2 mm	1.5	100–200 μm
NIR-II	5–10 mm	6.5	10–25 μm

### 2.2 Endoscopic assisted diagnosis

**FIGURE 3:**
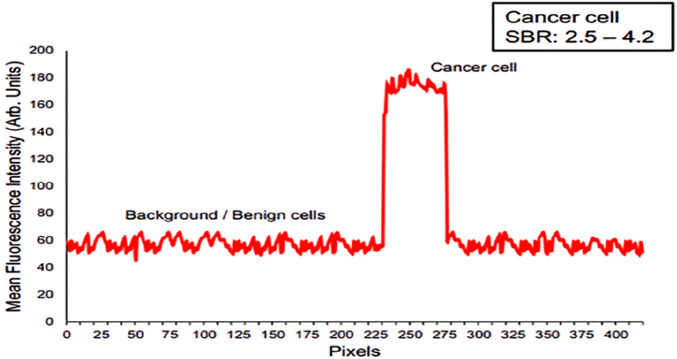
A cross-sectional fluorescence intensity profile of an individual malignant cell in NIR-nCLE images demonstrates a high SBR for cancer cells, with values ranging from 2.5 to 4.2. Copyright © 2022, Gregory T. Kennedy et al.

Integrating NIR-II imaging with bronchoscopic platforms or with needle-based confocal laser endomicroscopy (nCLE) enables the detection of abnormal molecular signals before overt morphological changes, thereby improving the detection of early-stage and certain intermediate-stage lung cancers (Chen et al., 2015). NIR-nCLE employs targeted NIR dyes to discriminate tumor from normal tissue at the cellular level and permits real-time identification of malignant cells during biopsy (Kennedy et al., 2022b). Kennedy et al. demonstrated that tumor specimens exhibited significantly higher mean fluorescence intensity than adjacent normal tissue (Figure 3) and that the method could detect single cancer cells at a 1:1,000 dilution. For nodules smaller than 2 cm, sensitivity and specificity were 100% and 92%, respectively, yielding an overall diagnostic accuracy of 96% (Kennedy et al., 2022a). The use of fluorescent contrast agents further enhances molecular targeting and early-detection sensitivity. Driven by increasing demands for precision and autonomy in robotic-assisted procedures, NIR imaging has been extended in endoscopic systems. Wei et al. developed a multidimensional light-field endoscope capable of acquiring trichromatic (RGB) images from two viewing angles with differing polarization states, enabling three-dimensional color reconstruction and polarization analysis, while concurrently capturing ICG NIR fluorescence without an additional “electronic staining” step. Combined with specific fluorescent probes to discriminate tumor from inflammatory tissue, this integrated approach markedly improves the accuracy and specificity of endoscopy-assisted diagnosis (Charanya et al., 2014; Wei et al., 2025).

### 2.3 The auxiliary application of liquid biopsy

**FIGURE 4:**
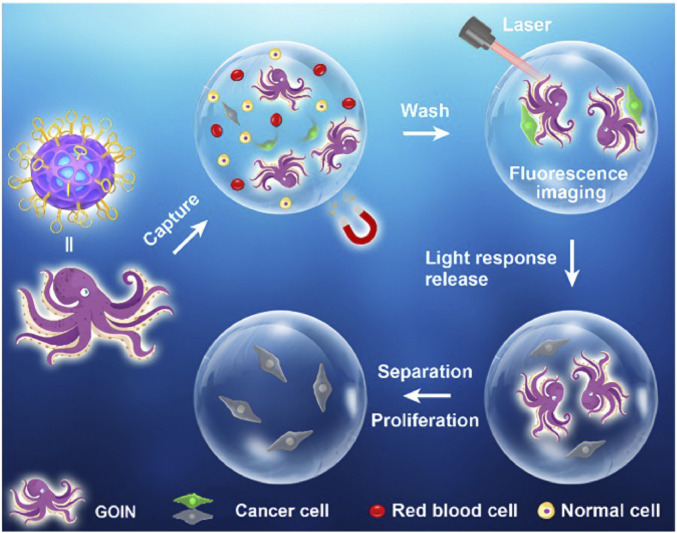
Schematic illustration of the structure and application of GOIN for CTC capturing, Separation, Imaging, and NIR-Triggered Release. Copyright © 2023 American chemical society.

Monitoring circulating tumor cells (CTCs) is critical for evaluating tumor progression and treatment response; however, many current capture–release techniques depend on disruptive processes that limit reusability and can compromise CTC viability (Chen et al., 2019; Vandghanooni et al., 2022). To overcome these limitations, Gao et al. developed a magnetic, fluorescent covalent organic framework (COF)–based “glowing octopus” nanomachine (GOIN) functionalized with the AS1411 aptamer for selective CTC capture, fluorescence imaging, magnetic separation, and NIR-triggered thermal release. Importantly, GOIN preserves cell proliferative capacity after release, offering a promising approach for repeatable liquid biopsy workflows (Chen et al., 2021; Gao et al., 2023; Gao et al., 2024; Li C. H. et al., 2021) (Figure 4). Despite such advances, the extremely low frequency of CTCs in early-stage cancer patients continues to pose a sensitivity bottleneck for detection (Ding et al., 2018; Dong et al., 2018; Su et al., 2019). Addressing this challenge, Xiang et al. engineered an NIR-activatable DNA nanodevice that selectively binds survivin (BIRC5) mRNA in tumor cells and locally releases antisense oligonucleotides (ASOs). The device performs NIR-to-UV upconversion, using the generated UV emission to corroborate target mRNA presence, thereby enhancing liquid-biopsy detection rates and improving the sensitivity and diagnostic accuracy of gene-therapy monitoring (Miaomiao et al., 2024).

### 2.4 Multimodal imaging and targeted probes

**FIGURE 5:**
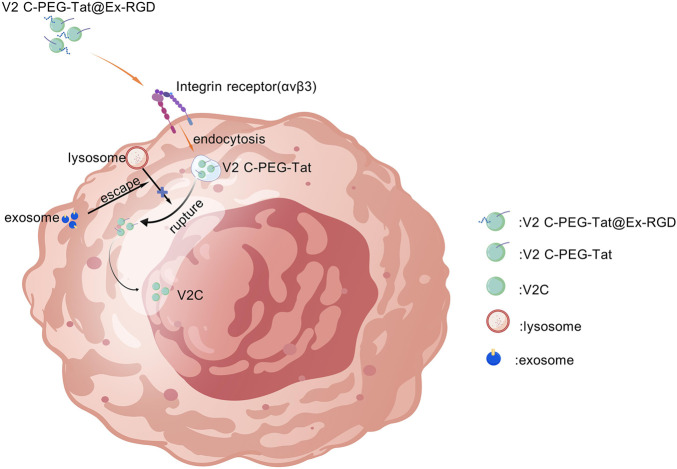
Tat peptide-mediated transport delivers V2C QDs across the nuclear pore complex (NPC) into the nucleus. Imaging revealed an enhanced perinuclear signal. Subsequent NIR-II laser irradiation within the nucleus produced localized hyperthermia that directly damages nuclear DNA and other essential nuclear biomolecules.

Single-modality optical imaging lacks sufficient anatomical context and quantitative information; consequently, multimodal strategies that combine NIR-II with complementary modalities such as photoacoustic (PA) imaging, CT, or MRI can integrate the strengths of each technique to enhance diagnostic performance (He et al., 2018; Li et al., 2019b; Li et al., 2021b; Wang et al., 2019; Wu M. et al., 2021). Numerous NIR-II fluorophores are inherently amenable to multimodal implementation, producing fluorescence, PA, or MR contrast from a single platform to enable synchronous imaging workflows (Cai et al., 2020; Cheng et al., 2017; Dai et al., 2017; Dong et al., 2020; Guo et al., 2019; Li et al., 2018; Li J. et al., 2019; Li et al., 2019b; Li S. et al., 2020; Li et al., 2021a; Liu et al., 2017; Okubo et al., 2021; Ren et al., 2018; Ren et al., 2020; Sun et al., 2020; Wu J. et al., 2021; Xu et al., 2019; Yang et al., 2017; Zhang Y. et al., 2020; Zhang Z. et al., 2020; Zhang X. et al., 2021; Zhao et al., 2019; Zheng et al., 2020a; Zheng et al., 2020b). For instance, Cheng et al. reported PEG-TONW NR, a probe with NIR-II absorption, CT and PA imaging capability, and high photothermal conversion efficiency for image-guided photothermal therapy (Cheng et al., 2019). Cao and co-workers synthesized V2C quantum dots that exhibit strong photothermal responses and multimodal imaging performance in the NIR-II window; exosome-mediated delivery enhanced nucleus-targeted photothermal therapy (PTT) efficacy (Figure 5) (Cao et al., 2019). Xu et al. described an aggregation-induced-emission (AIE) theranostic molecule, T-TTBMTD, featuring a donor–acceptor–donor (D–A–D) architecture that imposes a highly twisted conformation and extends NIR-II emission. T-TTBMTD–based NIR-II imaging enables tumor labeling and guides light-based therapies such as photodynamic therapy (PDT) or PTT. Joint fluorescence (FL), PA, and photothermal imaging permits identification and optimization of the therapeutic time window, thereby improving treatment outcomes (Xu D. et al., 2023).

Moreover, activatable probes that respond to overexpressed biomarkers or reactive species (e.g., H_2_O_2_) enable highly selective combined diagnostic and therapeutic interventions. To exploit tumor-elevated H_2_O_2_, Chen et al. synthesized an ER-targeted probe (TS-BOD-B) that is activated by H_2_O_2_ to produce amplified NIR fluorescence and photothermal signals for selective imaging and therapy (Chen et al., 2023). Zhang et al. developed a fluorescent probe (F-1) to target human neutrophil elastase (HNE) in A549 cells; F-1 demonstrated high selectivity, low cytotoxicity, and real-time, NIR turn-on fluorescence upon HNE activation, enabling sensitive enzymatic detection (Zhang W. et al., 2021). Xu et al. prepared an Nd@NaGdF4 probe conjugated with a ProGRP antibody, which exhibited low toxicity and enabled near-infrared fluorescence (NIRF) imaging of small-cell lung cancer (SCLC) cells and subcutaneous xenograft models for precise tumor identification; this approach offers a promising avenue for noninvasive SCLC diagnosis (Xu L. et al., 2023).

Notably, NIR-II multimodal imaging can be integrated with machine learning approaches to markedly enhance image quality via super-resolution reconstruction, denoising, and feature enhancement. Deep-learning semantic segmentation models (e.g., U-Net) can automatically delineate lesions in NIR-II images—such as tumors or vascular abnormalities—thereby reducing manual annotation errors and interobserver bias. Several studies have reported that AI systems coupled with NIR-II imaging accurately differentiate malignant foci from surrounding normal tissue in breast cancer specimens and can assist intraoperative margin assessment (Y. et al., 2023). Furthermore, combining targeted imaging probes with machine learning enables kinetic modeling of dynamic imaging data (e.g., probe accumulation and clearance). Such modeling supports real-time evaluation of nanomedicine delivery efficiency, tumor targeting, and therapeutic response, and provides quantitative guidance for individualized dosing strategies (Dunn et al., 2023). Looking ahead, imaging probes can be engineered as activatable sensors—responsive to microenvironmental pH or specific enzymatic activities—and machine-learning algorithms can analyze spatiotemporal activation patterns to enable dynamic disease monitoring and evaluation of treatment response (Shen et al., 2024).

## 3 Application of NIR-II in lung cancer treatment

### 3.1 Surgery

**FIGURE 6:**
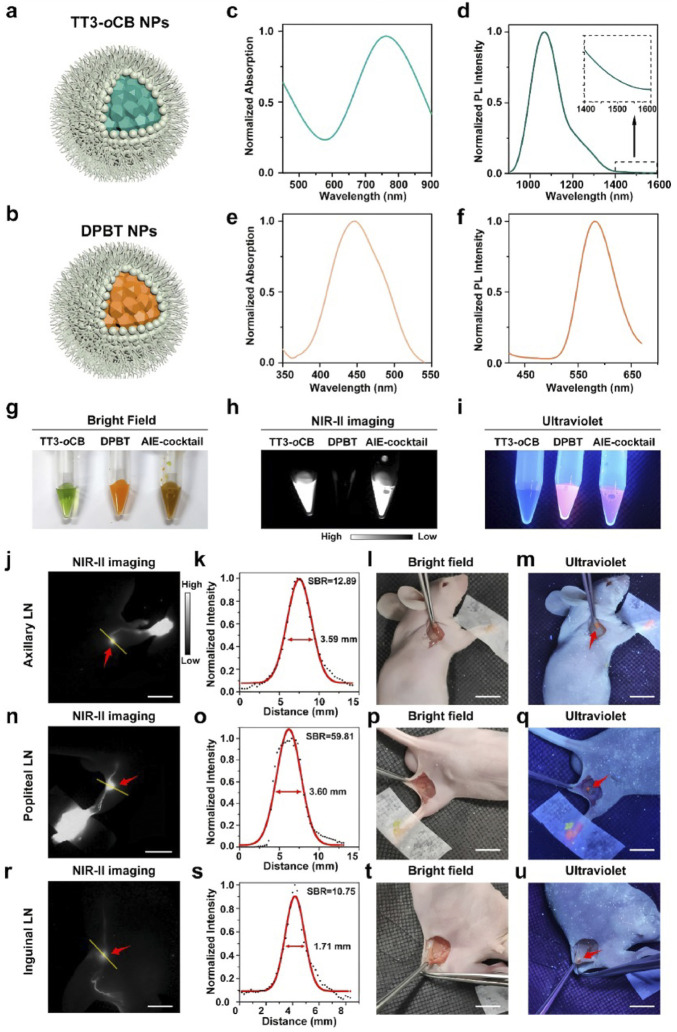
Schematic illustration of **(a)** TT3-oCB NPs and **(b)** DPBT NPs. **(c)** Absorption spectrum and **(d)** Photoluminescence (PL) spectrum of TT3-oCB NPs. **(e)** Absorption spectrum and **(f)** PL spectrum of DPBT NPs. Comparison of TT3-oCB NPs, DPBT NPs, and the AIE NPs cocktail **(g)** under bright field, **(h)** under the excitation of an 808 nm laser and **(i)** under the excitation of an ultraviolet lamp. NIR-II fluorescence images of **(j)** axillary lymph node, **(n)** popliteal lymph node and **(r)** inguinal lymph node of the nude mice treated with the AIE NPs cocktail (scale bar:1 cm). **(k,o,s)** Cross-sectional fluorescence intensity profiles (black) and the Gaussian fits along the yellow lines in **(j, n, r, l, p, t)** the pictures of the areas around the lymph nodes under bright field (scale bar:1 cm). Visible fluorescence signals of the **(m)** axillary lymph node, **(q)** popliteal lymph node and **(u)** inguinal lymph node under the ultraviolet lamp (scale bar:1 cm).© 2022 The Xiaoxiao Fan,Qiming Xia,Shunjie Liu,Zheng Zheng,Yiyin Zhang, Tianxiang Wu,Yixuan Li,Guping Tang,Ben Zhong Tang,Jun Qian,Hui Lin. Published by Elsevier Ltd.

Accurate determination of resection margins is essential for curative lung cancer surgery, yet conventional visual inspection and palpation frequently fail to delineate tumor boundaries with sufficient precision. Fluorescence-guided surgery has emerged as a key adjunct for achieving more precise resections. NIR-II fluorescence molecular imaging (FMI) markedly increases tumor-to-background contrast, thereby improving the likelihood of complete tumor removal (Lauwerends et al., 2021). Clinically, investigators have employed either nonspecific dyes (e.g., ICG) or tumor-targeted labels such as antibody-conjugated fluorophores. In an *ex vivo* study, Li et al. applied an EGFR-targeted IRDye800CW to suspicious lung specimens, reporting 85.7% sensitivity and 100% specificity for tumor detection, and favorable performance for nodal metastasis identification (sensitivity 77.8%, specificity 92.1%) (Li et al., 2023). Fan and colleagues introduced a composite navigation approach using a mixture of AIE nanoparticles to provide dual-mode intraoperative guidance (visible and NIR-II fluorescence). This bimodal configuration accelerates intraoperative imaging and is readily compatible with fluorescence endoscopy platforms, enhancing clinical translatability (Fan et al., 2022) (Figure 6).

Zhang et al. described an anthocyanin–albumin complex with promising *in vivo* imaging characteristics: chlorinated anthocyanin derivatives (e.g., 1-Cl, 5-Cl) localize selectively in lung tumor tissue while showing low uptake in normal lung, a selectivity attributed to upregulated albumin-binding or uptake mechanisms in tumor cells that facilitate complex internalization (YueweiZhang et al., 2023). Donor–acceptor–donor (D–A–D) small-molecule dyes represent another promising category of NIR-II emitters. Although CH1055 exemplifies this class, it is prone to excited-state quenching and chemical degradation, which limit its stability in organic matrices (Sun et al., 2016). To address these limitations, Li et al. developed p-FE, a nanoprobe built on an S–D–A–D–S (FE) scaffold that affords optical and chemical stabilization of D–A–D chromophores and reaches a quantum yield of ∼1.65%. Hybridizing p-FE with single-walled carbon nanotubes enables dual-channel NIR-II imaging of both the vasculature and tumors *in vivo* (Li et al., 2020). Integration of such probes with complementary imaging agents holds promise for intraoperative margin delineation and guidance of resection extent in lung cancer. Ultimately, NIR-II-guided intraoperative imaging may serve as a valuable complement to standard histopathology, reducing intraoperative decision times and improving the efficiency and accuracy of margin assessment (Zhang Z. et al., 2024).

NIR-II fluorescence–guided surgery provides real-time, high-contrast tumor visualization and has become a key adjunct to precise tumor resection. Smart, activatable NIR-II fluorophores leverage an off–on switching mechanism to increase imaging signal-to-noise ratio and specificity, outperforming ICG overall (China et al., 2019). According to the tumor-associated biomarkers or microenvironmental cues they target, these probes can be grouped into four core strategies: (i) ROS-activatable probes that restore fluorescence by exploiting elevated reactive oxygen species (e.g., H_2_O_2_) in tumor cells; (ii) enzyme-activatable probes that undergo off–on conversion upon substrate cleavage by overexpressed tumor enzymes (e.g., matrix metalloproteinases); (iii) RSS-activatable probes that respond to high levels of reductive sulfur species (e.g., H_2_S or glutathione) within the tumor microenvironment; and (iv) TME-activatable probes that trigger fluorescence in response to global microenvironmental features such as acidic pH or hypoxia. Collectively, these designs enable selective activation at tumor sites and markedly improve imaging contrast.

Constitutively “always-on” probes (e.g., ICG, methylene blue) exhibit short tumor retention, high background, and limited specificity, and they often require prolonged metabolism to generate usable contrast—limitations that hinder real-time, precision surgery. In contrast, single-biomarker activatable probes frequently yield false positives in normal or inflamed tissues, undermining diagnostic accuracy. To overcome these limitations, Dou et al. (2025) reported HN-PBA, an NIR-II fluorescent probe employing dual, cascade activation by metabolic acidity and H_2_O_2_ for intraoperative real-time navigation and precise resection of both primary tumors and metastases. HN-PBA implements a dual-locking design in which a boronate ester responds to H_2_O_2_ and a carboxyrhodamine unit responds to acidity; only when both cues coexist is intramolecular charge transfer restored and NIR-II fluorescence switched on. In preclinical models, HN-PBA demonstrated high sensitivity and selectivity in the tumor microenvironment, enabled high-contrast imaging in subcutaneous, orthotopic, and lung metastasis models (tumor-to-normal ratio up to 27.5), and guided resection of microlesions as small as 0.25 mm. Collectively, these findings suggest that, relative to single-target activation, multi-target cascade activation further improves specificity and lowers false-positive rates.

**FIGURE 7:**
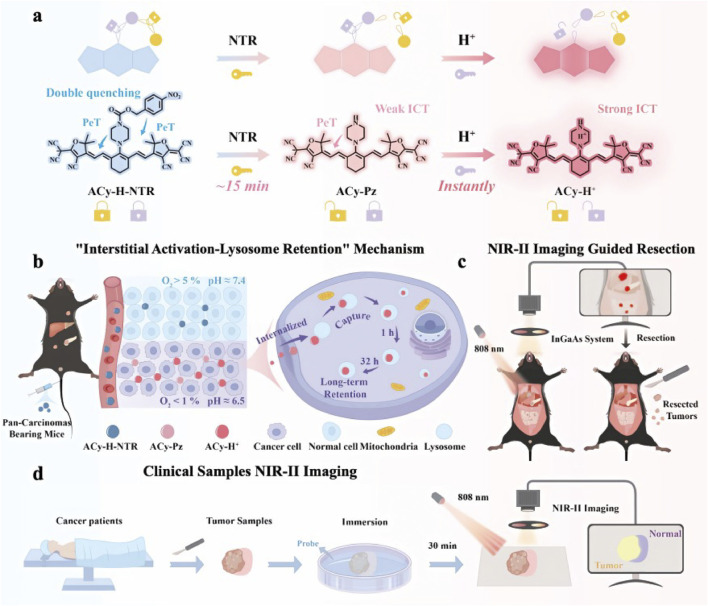
**(a)** Schematic diagram of the design of the tandem-locked and dual-quenched probe ACy-H-NTR, utilizing hypoxic and acidic microenvironments as universal biomarkers of malignant tumors. **(b)** Schematic illustration of the probe’s rapid response in the interstitial tissue and its long-term retention effect in lysosomes. **(c)** NIR-II imaging-guided resection in pan-carcinomas mouse models. **(d)** NIR-II imaging process for clinical cancer samples. © 2025 Wiley-VCH GmbH.

To overcome the slow response, limited sensitivity, and narrow tumor applicability of conventional dual-locking probes, Zhou et al. (2025) reported ACy-H-NTR, an NIR-II fluorescent probe that integrates tandem locking with dual quenching to enable high-contrast imaging and intraoperative navigation across multiple tumor types. ACy-H-NTR is activated in a cascade manner by sequential cues in the tumor microenvironment: (i) nitroreductase (NTR) under hypoxia reduces the p-nitrobenzyl trigger to release the intermediate ACy-Pz; and (ii) subsequent protonation of a piperazine moiety under acidic conditions restores and amplifies NIR-II fluorescence. (Figure 7).In preclinical models of pancreatic cancer, liver metastasis, and peritoneal metastasis, ACy-H-NTR delivered highly specific imaging with tumor-to-normal ratios of 7.8–16.8 and enabled precise resection of micrometastases <2 mm. By leveraging a synergistic tandem-locking–dual-quenching–lysosomal-retention design, the probe achieves enhanced activation efficiency and tumor selectivity, offering a promising molecular tool for pan-cancer, real-time intraoperative imaging and precision surgery.

Feng et al. (2025) fabricated AlGaN-based deep-ultraviolet (UVC) micro-LEDs and high-density arrays with lateral dimensions down to 3 μm. These sources enable photoresist exposure within seconds and micron-scale pattern transfer, offering a flexible, low-cost, and environmentally friendly alternative for lithography illumination. This advance could accelerate the micro/nanofabrication and integration of NIR-related imaging displays, indirectly driving the miniaturization and performance gains of intraoperative imaging systems.

### 3.2 Immunotherapy

**FIGURE 8:**
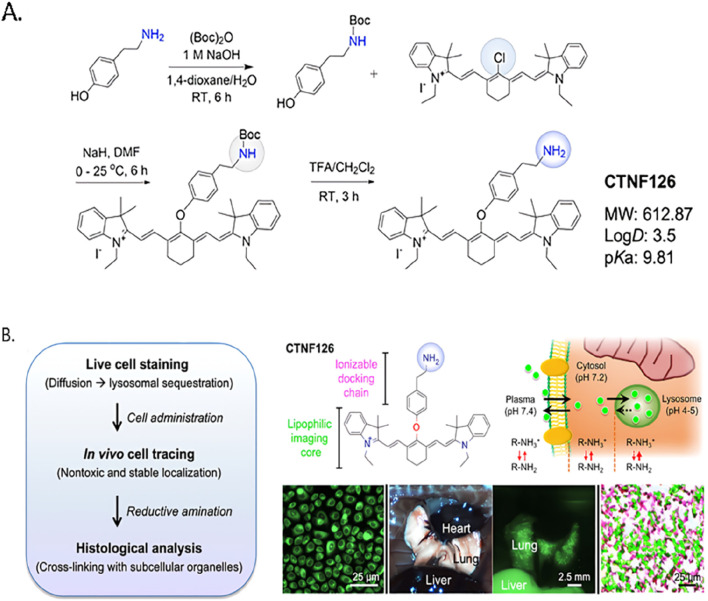
**(A)** Synthetic scheme of CTNF126 assembled from two functional moieties.**(B)** Longitudinal cellular tracking of the NIR fluorophore and lysosomal chelation of CTNF126. The thickness of the red arrows indicates the degree of ionization. Copyright© 2019 WILEY-VCH Verlag GmbH & Co. KGaA, Weinheim.

In immunotherapy studies, NIR-II imaging is frequently employed to label and longitudinally track therapeutic immune populations (e.g., CD4^+^ and CD8^+^ T cells, natural killer (NK) cells, and dendritic cells) or immune agents, enabling monitoring of cellular biodistribution, homing, and therapeutic response (Fischbach et al., 2013; Kang et al., 2022; Restifo et al., 2012). Labeling approaches must minimize perturbation of cell phenotype and function; for instance, membrane dyes can impair migration, whereas mitochondrial stains may alter membrane potential and cellular metabolism (Frye and Edidin, 1970; Lulevich et al., 2009). To address these concerns, Choi and Henary reported CTNF126, a lysosome-targeted NIR fluorophore synthesized by appending a primary-amine docking motif onto a heptamethine scaffold. Protonation of the primary amine facilitates membrane translocation, and subsequent intracellular formaldehyde-mediated reductive amination rapidly fixes and sequesters the dye, reducing label transfer and preserving cell function (Figure 8) (Kim et al., 2020; Kim et al., 2021; Park et al., 2019).

An alternative strategy employs the NHS-ester form of fluorophores to covalently label cell-surface proteins; Mellanby et al. developed CIR38M, a tricarbocyanine-derived NIR probe that functions as a nontransferable, persistent label for noninvasive *in vivo* tracking of therapeutic T cells (Mellanby et al., 2018). NIR fluorophore-loaded nanoparticles have likewise been explored for *ex vivo* cell labeling, typically via endocytic uptake. Fuller et al. showed that silica nanoparticles coated with cationic polymers can mediate endosomal escape and deliver cargo to the cytosol (Fuller et al., 2008). Zhao et al. used NIR-activatable, phthalocyanine-loaded lipid nanoparticles to label bone marrow-derived macrophages (BMMs); upon NIR stimulation, these engineered BMMs release chemotherapeutic payloads that ablate primary tumors and elicit immunogenic cell death (Huang et al., 2021). Similarly, Lim and colleagues labeled human NK cells with antibody-conjugated fluorescent quantum dots to visualize the intratumoral behavior and therapeutic impact of injected NK cells by NIR imaging (Lim et al., 2009). Overall, NIR-II imaging provides a powerful platform for evaluating the distribution and efficacy of cell-based therapies and for informing optimization of immunotherapeutic strategies.

### 3.3 PDT

Photodynamic therapy (PDT) is characterized by a favorable safety profile, light-triggered activation, and limited systemic toxicity (Agostinis et al., 2011; Xie et al., 2021). PDT exerts antitumor effects primarily through the generation of reactive oxygen species (ROS), which inflict mitochondrial damage and thereby disrupt reprogrammed metabolic pathways in cancer cells (Jia et al., 2022; Li G. et al., 2021). Concurrent inhibition of cellular production of reducing equivalents—for example, via 2-deoxy-D-glucose (2-DG)-mediated suppression of NADPH synthesis—alters mitochondrial redox balance and reduces antioxidant capacity, rendering mitochondria more vulnerable to ROS-mediated injury and thereby potentiating PDT efficacy (Sies et al., 2022).

**FIGURE 9:**
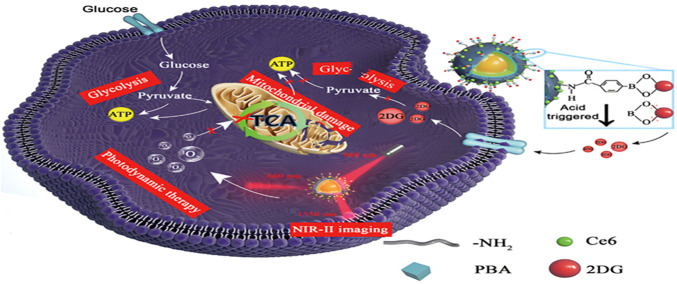
Proposed mechanism of action of LnNP@mSiO2-GC in tumor therapy: upconverted 650-nm emission activates Ce6 to produce singlet oxygen (1O2) for PDT. The generated 1O2 suppresses expression of hexokinase 2 (HK2) and lactate dehydrogenase A (LDHA), thereby impairing mitochondrial function and potentiating the anti-glycolytic activity of 2DG. Consequently, glycolytic metabolic reprogramming is disrupted and the efficacy of starvation-based therapy is substantially enhanced. Copyright© 2024 Science China Press. Published by Elsevier B.V. and Science China Press. All rights reserved.

Building on this rationale, Wu et al. reported LnNP@mSiO_2_-GC nanoparticles that combine NIR activated starvation therapy with PDT (Wu X. et al., 2024). The nanoparticles release 2-DG in response to the acidic tumor microenvironment. Upon 980 nm irradiation, glycolytic inhibition by 2-DG is coupled with Er3^+^-mediated upconversion emission at ∼650 nm, which activates Ce6 to generate singlet oxygen (^1^O_2_). The resulting mitochondrial damage synergizes with metabolic starvation to induce tumor cell death (Figure 9). By converting NIR to red emission under laser irradiation, LnNP@mSiO_2_-GC enables controlled ROS production and effectively bypasses compensatory flux through the tricarboxylic acid (TCA) cycle, improving therapeutic outcomes.

Transition-metal complexes are also widely exploited in PDT because of their tunable electronic structures and favorable photophysical and catalytic properties. He et al. synthesized a ruthenium complex (RU-I) incorporating a highly conjugated indole-derived ligand, yielding an absorption red shift of ≈100 nm and a strong absorption band centered at 660 nm (He et al., 2021). RU-I accumulates in lysosomes and, upon red-light exposure, disrupts lysosomal integrity and function, thereby promoting cancer cell death. Separately, Zhang et al. engineered nucleic-acid-modified UiO-66-NH2 metal–organic frameworks (NMOFs) loaded with Zn(II) protoporphyrin IX (ZnPPIX) as a photosensitizer (Zhang P. et al., 2022). In this design, Zr^4+^ nodes coordinate with two distinct hairpin DNA scaffolds containing microRNA recognition sequences to enable miRNA-triggered selective imaging; visible-light irradiation then induces ROS-dependent tumor cell killing. Collectively, these strategies—combining metabolic modulation, NIR-activated upconversion, and metal-based photosensitizers—offer complementary routes to enhance PDT potency and selectivity.

### 3.4 PTT

Photothermal therapy (PTT) is a principal modality of phototherapy that induces tumor cell injury, necrosis, or thermal ablation by disrupting intracellular bioactive molecules; it is valued for being minimally invasive, highly selective, and associated with limited systemic toxicity (Li et al., 2022; Zhang Y. et al., 2022). Photothermal agents (PTAs), the active constituents of PTT, are frequently incorporated into nanoparticle (NP) platforms to facilitate targeted delivery and controlled photothermal conversion (Chen et al., 2020; Lan et al., 2023).

**FIGURE 10:**
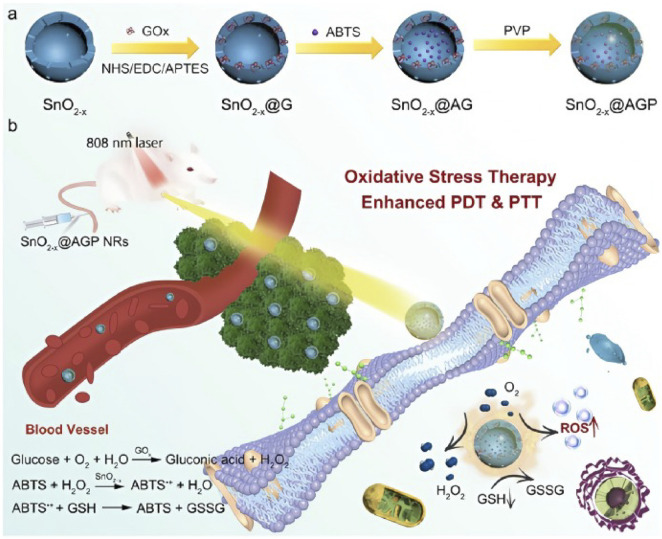
**(a)** schematic illustration to show the key steps involved in the preparation of hollow mesoporous SnO2−x@AGPNanoreactors and **(b)** associated major mechanistic pathways in cancer therapy. Copyright © 2023, American chemical society.

Dong et al. synthesized a croconaine-derived conjugate (CR-TPE-T) that self-assembles into nanoparticles with strong NIR absorption, high photothermal conversion efficiency, good photostability, and favorable biocompatibility (Dong et al., 2024). These CR NPs demonstrate efficient tumor accumulation in the NIR-II window and mediate tumor ablation under NIR-II guided PTT using 808 nm laser irradiation. Feng et al. developed a tumor-microenvironment-responsive, NIR-activatable theranostic nanoreactor (SnO_2_−x@AGP), in which hollow mesoporous SnO_2_−x functions both as a carrier and as an active therapeutic component: its abundant oxygen vacancies enhance PDT/PTT activity. The system consumes intratumoral glucose to elevate endogenous H_2_O_2_ and thereby induce cytotoxic oxidative stress—a strategy referred to as oxidative-stress therapy—rather than relying solely on energy deprivation (Feng et al., 2023) (Figure 10). Ge and colleagues reported a gold-nanocluster–based probe, Au^44^MBA26-Cy7, which leverages AIE to boost Au^44^ NC photoluminescence in the NIR-II region. Concurrent local enrichment of Cyanine-7 (Cy7) on the cluster surface increases photothermal conversion efficiency to ∼65.1%, substantially enhancing PTT performance (Yang et al., 2023).

### 3.5 ADC

**FIGURE 11:**
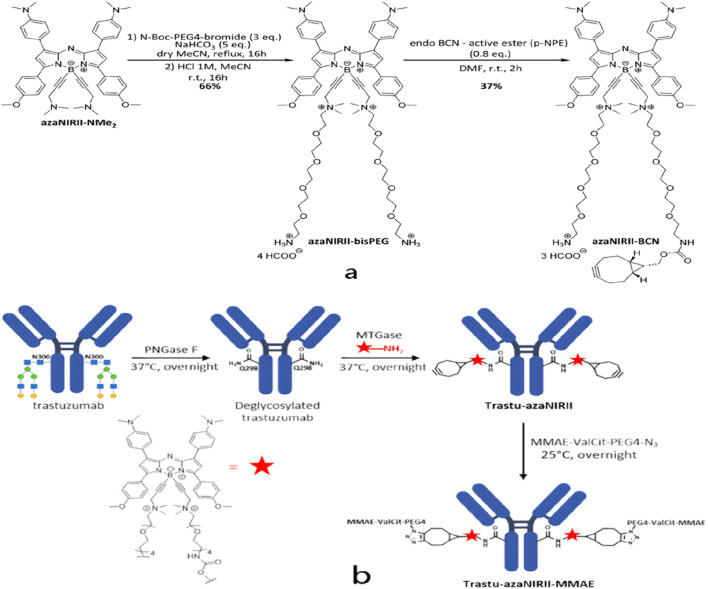
**(a)** Synthesis of the azaNIR-II-BCN platform.**(b)** Synthesis of the fluorescent ADC Trastu–azaNIR-II–MMAE.Copyright© 2025, American chemical society.

Real-time visualization of antibody–drug conjugates (ADCs) offers critical information on tumor-targeting specificity, therapeutic efficacy, and potential off-target accumulation that can precipitate adverse events. To facilitate accurate *in vivo* tracking, Chazeau et al. developed a modular fluorescent platform based on a NIR-II-emitting aza-BODIPY scaffold that can be site-specifically appended to IgG1 antibodies to produce well-defined fluorescent ADCs (Figure 11) (Chazeau et al., 2025). Using this approach, the authors generated a HER2-targeted trastuzumab conjugate incorporating the NIR-II aza-BODIPY motif; subsequent installation of a cytotoxic payload yielded fluorescent ADCs that exhibited significant antitumor activity in preclinical evaluation.

Thankarajan and colleagues reported a complementary dual-dye labeling strategy (IRDAbmXCyCLB) and validated its performance in HER2-positive BT-474 xenograft models (Thankarajan et al., 2021). This dual-dye system enabled quantitative, longitudinal monitoring of drug release and proved robust against variations in experimental conditions and inter-animal response heterogeneity. Collectively, these studies underscore the translational potential of NIR-labeled ADCs for HER2-positive lung cancers.

Beyond HER2, NIR-based ADC strategies have been applied to other clinically relevant cell-surface receptors—including epidermal growth factor receptor (EGFR), prostate-specific membrane antigen (PSMA), folate receptor, and cancer antigen 125 (CA-125) (Goff et al., 1994; Wei et al., 2022)—as well as selected intracellular targets (Wang et al., 2015). Integrating NIR imaging into ADC design can improve targeted delivery efficiency, enable precise spatiotemporal control of payload release, and optimize pharmacokinetic and targeting properties, thereby broadening the scope for combined diagnostic and therapeutic (theranostic) applications in malignancies such as lung cancer (Godard et al., 2023; Molecular Imaging Program, 2019; Nagaya et al., 2015; Sato et al., 2018).

## 4 Integrated diagnosis and treatment

The central principle of NIR-enabled theranostics is to integrate diagnostic imaging and therapeutic modalities through nanotechnology, exploiting the deep-tissue penetration of NIR light for precise localization, real-time monitoring, and immunomodulation, while employing tumor-microenvironment-responsive designs to enhance specificity and retention. In lung cancer, NIR-based theranostic strategies couple imaging guidance, targeted NIR photoimmunotherapy (NIR-PIT), and multimodal regimens—for example, combinations of immunotherapy, chemotherapy, and photothermal therapy—to achieve a seamless transition from accurate diagnosis to effective treatment.

Notably, NIR-II fluorescence guidance has enabled integrated multimodal interventions: NIR-IIb imaging can direct combined surgery, metabolic-starvation therapy, and chemodynamic therapy (CDT) for synchronous diagnostic–therapeutic action *in vivo* (Han et al., 2023). Mitochondria-targeted NIR aggregation-induced-emission (AIE) photosensitizers serve dual roles in high-contrast imaging and singlet-oxygen/reactive-oxygen-species (ROS) generation for precision photodynamic therapy (PDT). These agents display high tumor specificity and immunogenic potential, minimizing collateral damage to normal tissues (Chen et al., 2024) and inducing immunogenic cell death (ICD) that promotes durable antitumor immunity (Ogawa et al., 2017).

To accelerate clinical translation, key challenges must be addressed, including development of multi-targeted synergistic platforms, standardization of therapeutic dosing and illumination protocols, and validation across heterogeneous and clinically relevant tumor models (Georgieva et al., 2021; Kato et al., 2021). Addressing these gaps will be essential to realize the full potential of NIR-guided theranostics in malignant lung disease.

## 5 Prospects and challenges

NIR light, particularly in the NIR-II window, offers superior tissue penetration compared with visible wavelengths, making it well suited for noninvasive and minimally invasive diagnostics, such as tissue oxygenation monitoring and ischemia detection. Advanced NIR probes—for example, NIR-emissive polymeric nanovesicles—substantially enhance imaging sensitivity and are driving progress in molecular diagnostics and PDT (Bon and Cognet, 2022; Ghoroghchian et al., 2009). Complementary advances in theoretical modeling and spectral prediction further improve NIR spectral analysis and facilitate the integration of multimodal imaging techniques (Beć and Huck, 2019). Emerging NIR-II–visualizable gas-based therapies, for example, controlled-release nanoplatforms for carbon monoxide (CO) or hydrogen sulfide (H_2_S), permit real-time imaging of gas accumulation and release within tumors. These approaches can improve antitumor efficacy while reducing systemic toxicity and are particularly promising for deep-seated or metastatic lung cancers (Wu J. et al., 2024). Despite these advances and the substantial innovations that NIR imaging has introduced to cancer diagnosis and treatment, practical performance remains constrained by detector sensitivity and noise, which limit effective penetration depth and spatial resolution (Deriu et al., 2023). Current NIR-II organic probes (e.g., donor–acceptor–donor [D–A–D] molecules) suffer from low quantum yields, poor aqueous solubility, and slow clearance, whereas inorganic probes (e.g., quantum dots) raise concerns about long-term toxicity and metal-ion leaching (Dai et al., 2021a) (Table 2). Future progress will require the development of probes that combine high brightness, biodegradability, and targeting specificity, together with strategies to improve tissue penetration (for example, reduced particle size and enhanced targeting ligands) and computational solutions for motion-artifact correction (Zhang J. et al., 2024).

**TABLE 2:** Current advantages and limitations of NIR-II imaging for lung cancer applications.

Category	Advantage	Limitation
Imaging performance	• Deep-tissue penetration capability (NIR-II) • High spatial resolution and signal-to-background ratio • Low tissue scattering and low autofluorescence • Radiation-free with straightforward operation • Supports real-time dynamic imaging and intraoperative navigation	• Limited penetration depth and resolution due to detector performance limitations • Motion artifacts impair image quality • Low quantum yield and poor water solubility of existing probes
Diagnostic application	• Sensitive imaging of tumor microenvironmental features, such as hypoxia and acidosis • Endoscopic guidance for early lesion detection (e.g., NIR-nCLE) • Capture and imaging of CTCs in liquid biopsy • Advantages of multimodal imaging fusion (FL/PA/CT/MRI) • AI-augmented image analysis for enhanced lesion segmentation, classification, and detection	• The scarce presence of CTCs in early lung cancer results in limited assay sensitivity • Slow metabolic clearance of probes may result in prolonged *in vivo* retention • Certain imaging agents raise biocompatibility and biosafety concerns
Therapeutic application	• Fluorescence-guided surgery (FGS) to improve resection precision and margin assessment • Light-activated PTT/PDT enables localized cytotoxicity with spatiotemporal control • *In vivo* tracking of immune cells for monitoring therapy response and cell-based treatments • Imaging-guided evaluation of ADC delivery, tumor uptake and drug release kinetics • Theranostic platforms integrating diagnostic imaging and therapeutic functions for personalized oncology	• Organic fluorescent probes suffer from rapid photobleaching under repeated excitation • Inorganic probes such as quantum dots raise concerns regarding long-term toxicity and bioaccumulation • Therapeutic dosing and light-delivery parameters lack standardization across preclinical and clinical studies
Probes and materials	• Microenvironment-responsive probes (e.g., pH- or enzyme-activated) for stimulus-triggered signal or payload release • Integrated NIR-II/PA/MRI agents enable deep-tissue visualization with both high sensitivity and high spatial resolution • AIE-based probes and upconversion materials enable high photostability, anti-photobleaching and deep-tissue excitation • Receptor-targeted probes (e.g., anti-EGFR, anti-HER2, folate conjugates) enhance tumor uptake and diagnostic specificity	• Organic D–A–D probes typically require complex multistep synthesis and exhibit poor aqueous solubility • Risk of metal ion leaching (e.g., from quantum dots) • Large-scale manufacturing and clinical translation remain challenging
Clinical translation and system integration	• Integration of robotic surgery with AI to enhance surgical precision • Emerging directions such as visualization-guided gas therapies • Interdisciplinary collaboration to drive technological innovation	• Lack of standardized operating procedures and clinical validation • Limited adaptability to complex tumor models • High costs and heavy reliance on specialized equipment

Moreover, advancing NIR-II technology calls for close integration of materials science, imaging science, and clinical research; for instance, engineering tumor-responsive “activatable” probes (sensitive to pH or enzymatic activities) will demand interdisciplinary efforts to overcome design complexity and enable scalable manufacturing (Zhang Y. et al., 2021).

For multi-target activation, implementing AND-gate responses to multiple biomarkers (e.g., ROS and specific enzymes) substantially enhances diagnostic specificity in complex diseases and has become a key research direction. Reversible systems remain in an exploratory stage; the central task is to design molecular switches that reversibly report dynamic physiological cues (e.g., pH or redox fluctuations), enabling continuous monitoring of disease progression and assessment of therapeutic response. *In situ* labeling—especially bioorthogonal click chemistry—builds fluorescent signals directly within lesions *in vivo*, affording exceptional spatial resolution and localization accuracy, and thus represents an ideal tool for precision applications such as surgical navigation (Fu et al., 2025). Beyond simply appending antibodies or peptides as targeting ligands, greater gains in targeting and specificity arise from coupling targeted delivery with microenvironment-triggered activation—for instance, leveraging tumor acidity to unmask reactive groups, thereby amplifying signals selectively at the lesion and improving imaging signal-to-noise and therapeutic selectivity. Collectively, the integrated application of these strategies could accelerate the clinical translation of next-generation intelligent activatable probes.

## References

[B1] Agostinis P. Berg K. Cengel K. A. Foster T. H. Girotti A. W. Gollnick S. O. (2011). Photodynamic therapy of cancer: an update. CA Cancer J. Clin. 61 (4), 250–281. 10.3322/caac.20114 21617154 PMC3209659

[B2] Beć K. B. Huck C. W. (2019). Breakthrough potential in near-infrared spectroscopy: spectra simulation. A review of recent developments. Front. Chem. 7, 48. 10.3389/fchem.2019.00048 30854368 PMC6396078

[B3] Bon P. Cognet L. (2022). On some current challenges in high-resolution optical bioimaging. ACS Photonics 9 (8), 2538–2546. 10.1021/acsphotonics.2c00606 35996373 PMC9389608

[B4] Cai W. Sun J. Sun Y. Zhao X. Guo C. Dong J. (2020). NIR-II FL/PA dual-modal imaging long-term tracking of human umbilical cord-derived mesenchymal stem cells labeled with melanin nanoparticles and visible HUMSC-based liver regeneration for acute liver failure. Biomater. Sci. 8 (23), 6592–6602. 10.1039/d0bm01221a 33231594

[B5] Cao Y. Wu T. Zhang K. Meng X. Dai W. Wang D. (2019). Engineered exosome-mediated Near-Infrared-II region V(2)C quantum dot delivery for nucleus-target low-temperature photothermal therapy. ACS Nano 13 (2), 1499–1510. 10.1021/acsnano.8b07224 30677286

[B6] Charanya T. York T. Bloch S. Sudlow G. Liang K. Garcia M. (2014). Trimodal color-fluorescence-polarization endoscopy aided by a tumor selective molecular probe accurately detects flat lesions in colitis-associated cancer. J. Biomed. Opt. 19 (12), 126002. 10.1117/1.Jbo.19.12.126002 25473883 PMC4255434

[B7] Chazeau E. Pipier A. Wegner K. D. Ghiringhelli F. Sancey L. Paul C. (2025). NIR-II aza-BODIPY platform for the development of a fluorescent antibody drug conjugate. J. Med. Chem. 68 (7), 7232–7242. 10.1021/acs.jmedchem.4c02777 40152348

[B9] Chen H. Lin Z. Mo L. Wu T. Tan C. (2015). Near-infrared spectroscopy as a diagnostic tool for distinguishing between normal and malignant colorectal tissues. Biomed. Res. Int. 2015, 472197. 10.1155/2015/472197 25654106 PMC4309295

[B10] Chen Y. Tyagi D. Lyu M. Carrier A. J. Nganou C. Youden B. (2019). Regenerative NanoOctopus based on multivalent-aptamer-functionalized magnetic microparticles for effective cell capture in whole blood. Anal. Chem. 91 (6), 4017–4022. 10.1021/acs.analchem.8b05432 30649851

[B11] Chen Y. Gao Y. Chen Y. Liu L. Mo A. Peng Q. (2020). Nanomaterials-based photothermal therapy and its potentials in antibacterial treatment. J. Control Release 328, 251–262. 10.1016/j.jconrel.2020.08.055 32889053

[B12] Chen Y. Gao P. Pan W. Shi M. Liu S. Li N. (2021). Polyvalent spherical aptamer engineered macrophages: X-ray-actuated phenotypic transformation for tumor immunotherapy. Chem. Sci. 12 (41), 13817–13824. 10.1039/d1sc03997k 34760167 PMC8549783

[B13] Chen R. Wang R. Sun J. Dong X. Dong C. Sun L. (2023). An activatable endoplasmic reticulum-targeted probe for NIR imaging-guided photothermal therapy. Org. Biomol. Chem. 21 (29), 5919–5923. 10.1039/d3ob00782k 37435862

[B14] Chen X. Li Y. Su J. Zhang L. Liu H. (2024). Progression in near-infrared fluorescence imaging technology for lung cancer management. Biosens. (Basel) 14 (10), 501. 10.3390/bios14100501 39451714 PMC11506746

[B15] Cheng K. Chen H. Jenkins C. H. Zhang G. Zhao W. Zhang Z. (2017). Synthesis, characterization, and biomedical applications of a targeted dual-modal Near-Infrared-II fluorescence and photoacoustic imaging nanoprobe. ACS Nano 11 (12), 12276–12291. 10.1021/acsnano.7b05966 29202225

[B16] Cheng Y. Yang F. Xiang G. Zhang K. Cao Y. Wang D. (2019). Ultrathin tellurium oxide/ammonium tungsten bronze nanoribbon for multimodality imaging and second near-infrared region photothermal therapy. Nano Lett. 19 (2), 1179–1189. 10.1021/acs.nanolett.8b04618 30640481

[B17] China K. L. F. E. N. T. U. N. Electronics K. L. O. Displays I. Posts J. K. L. B. N. U. China T. N. China K. L. F. E. N. T. U. N. (2019). Recent advances on activatable NIR‐II fluorescence probes for biomedical imaging. Adv. Opt. Mater. 7 (21), 1900917. 10.1002/adom.201900917

[B18] Choe R. Konecky S. D. Corlu A. Lee K. Durduran T. Busch D. R. (2009). Differentiation of benign and malignant breast tumors by *in-vivo* three-dimensional parallel-plate diffuse optical tomography. J. Biomed. Opt. 14 (2), 024020. 10.1117/1.3103325 19405750 PMC2782703

[B19] Dai Y. Yang D. Yu D. Cao C. Wang Q. Xie S. (2017). Mussel-inspired polydopamine-coated lanthanide nanoparticles for NIR-II/CT dual imaging and photothermal therapy. ACS Appl. Mater Interfaces 9 (32), 26674–26683. 10.1021/acsami.7b06109 28726368

[B20] Dai H. Shen Q. Shao J. Wang W. Gao F. Dong X. (2021a). Small molecular NIR-II fluorophores for cancer phototheranostics. Innov. (Camb) 2 (1), 100082. 10.1016/j.xinn.2021.100082 34557737 PMC8454557

[B21] Dai H. Wang X. Shao J. Wang W. Mou X. Dong X. (2021b). NIR-II organic nanotheranostics for precision oncotherapy. Small 17 (44), e2102646. 10.1002/smll.202102646 34382346

[B22] Deriu C. Thakur S. Tammaro O. Fabris L. (2023). Challenges and opportunities for SERS in the infrared: materials and methods. Nanoscale Adv. 5 (8), 2132–2166. 10.1039/d2na00930g 37056617 PMC10089128

[B23] Diao S. Blackburn J. L. Hong G. Antaris A. L. Chang J. Wu J. Z. (2015). Fluorescence imaging *in vivo* at wavelengths beyond 1500 nm. Angew. Chem. Int. Ed. Engl. 54 (49), 14758–14762. 10.1002/anie.201507473 26460151

[B24] Ding C. Zhang C. Yin X. Cao X. Cai M. Xian Y. (2018). Near-infrared fluorescent Ag(2)S nanodot-based signal amplification for efficient detection of circulating tumor cells. Anal. Chem. 90 (11), 6702–6709. 10.1021/acs.analchem.8b00514 29722265

[B25] Dong Z. Tang C. Zhao L. Xu J. Wu Y. Tang X. (2018). A microwell-assisted multiaptamer immunomagnetic platform for capture and genetic analysis of circulating tumor cells. Adv. Healthc. Mater 7 (24), e1801231. 10.1002/adhm.201801231 30565898

[B26] Dong Y. Dong S. Wang Z. Feng L. Sun Q. Chen G. (2020). Multimode imaging-guided photothermal/chemodynamic synergistic therapy nanoagent with a tumor microenvironment responded effect. ACS Appl. Mater Interfaces 12 (47), 52479–52491. 10.1021/acsami.0c17923 33196186

[B27] Dong Y. Wang H. Zhang X. Ding Y. Zou Y. Wang J. (2024). Croconaine-based NIR-II fluorescence imaging-guided tumor photothermal therapy induces long-term antitumor immune memory. J. Nanobiotechnology 22 (1), 481. 10.1186/s12951-024-02695-y 39135072 PMC11321165

[B28] Dou K. Lu J. Xing Y. Wang R. Won M. Kim J. (2025). Metabolic Acidity/H(2)O(2) dual-cascade-activatable molecular imaging platform toward metastatic breast tumor malignancy. Angew. Chem. Int. Ed. Engl. 64 (7), e202419191. 10.1002/anie.202419191 39511909

[B29] Dunn B. Hanafi M. Hummel J. Cressman J. R. Veneziano R. Chitnis P. V. (2023). NIR-II nanoprobes: a review of components-based approaches to next-generation bioimaging probes. Bioeng. (Basel) 10 (8), 954. 10.3390/bioengineering10080954 37627839 PMC10451329

[B30] Fan X. Xia Q. Liu S. Zheng Z. Zhang Y. Wu T. (2022). NIR-II and visible fluorescence hybrid imaging-guided surgery via aggregation-induced emission fluorophores cocktails. Mater Today Bio 16, 100399. 10.1016/j.mtbio.2022.100399 36052153 PMC9424606

[B31] Feng Z. Tang T. Wu T. Yu X. Zhang Y. Wang M. (2021). Perfecting and extending the near-infrared imaging window. Light Sci. Appl. 10 (1), 197. 10.1038/s41377-021-00628-0 34561416 PMC8463572

[B32] Feng L. Zhao R. Yang L. Liu B. Dong S. Qian C. (2023). Tumor-specific NIR-activatable nanoreactor for self-enhanced multimodal imaging and cancer phototherapy. ACS Nano 17, 1622–1637. 10.1021/acsnano.2c11470 36623255

[B33] Feng F. Liu Y. Zhang K. Yang H. Hyun B.-R. Xu K. (2025). High-power AlGaN deep-ultraviolet micro-light-emitting diode displays for maskless photolithography. Nat. Photonics 19 (1), 101–108. 10.1038/s41566-024-01551-7

[B34] Fischbach M. A. Bluestone J. A. Lim W. A. (2013). Cell-based therapeutics: the next pillar of medicine. Sci. Transl. Med. 5 (179), 179ps177. 10.1126/scitranslmed.3005568 23552369 PMC3772767

[B35] Forde P. M. Spicer J. Lu S. Provencio M. Mitsudomi T. Awad M. M. (2022). Neoadjuvant nivolumab plus chemotherapy in resectable lung cancer. N. Engl. J. Med. 386 (21), 1973–1985. 10.1056/NEJMoa2202170 35403841 PMC9844511

[B36] Frye L. D. Edidin M. (1970). The rapid intermixing of cell surface antigens after formation of mouse-human heterokaryons. J. Cell Sci. 7 (2), 319–335. 10.1242/jcs.7.2.319 4098863

[B37] Fu Y. Zhang X. Wu L. Wu M. James T. D. Zhang R. (2025). Bioorthogonally activated probes for precise fluorescence imaging. Chem. Soc. Rev. 54 (1), 201–265. 10.1039/d3cs00883e 39555968

[B38] Fuller J. E. Zugates G. T. Ferreira L. S. Ow H. S. Nguyen N. N. Wiesner U. B. (2008). Intracellular delivery of core-shell fluorescent silica nanoparticles. Biomaterials 29 (10), 1526–1532. 10.1016/j.biomaterials.2007.11.025 18096220

[B39] Gao P. Wei R. Chen Y. Li X. Pan W. Li N. (2023). Pt nanozyme-bridged covalent organic framework-aptamer nanoplatform for tumor targeted self-strengthening photocatalytic therapy. Biomaterials 297, 122109. 10.1016/j.biomaterials.2023.122109 37058901

[B40] Gao P. Li X. Wei R. Pan W. Li N. Tang B. (2024). Glowing octopus-inspired nanomachine: a versatile aptasensor for efficient capture, imaging, separation, and NIR-triggered release of cancer cells. Anal. Chem. 96 (1), 309–316. 10.1021/acs.analchem.3c04115 38108827

[B41] Georgieva M. Gospodinova Z. Keremidarska-Markova M. Kamenska T. Gencheva G. Krasteva N. (2021). PEGylated nanographene oxide in combination with near-infrared laser irradiation as a smart nanocarrier in Colon cancer targeted therapy. Pharmaceutics 13 (3), 424. 10.3390/pharmaceutics13030424 33809878 PMC8004270

[B42] Ghoroghchian P. P. Therien M. J. Hammer D. A. (2009). *In vivo* fluorescence imaging: a personal perspective. Wiley Interdiscip. Rev. Nanomed Nanobiotechnol 1 (2), 156–167. 10.1002/wnan.7 20049787 PMC3091504

[B43] Godard A. Kalot G. Privat M. Bendellaa M. Busser B. Wegner K. D. (2023). NIR-II Aza-BODIPY dyes bioconjugated to monoclonal antibody trastuzumab for selective imaging of HER2-Positive ovarian cancer. J. Med. Chem. 66 (7), 5185–5195. 10.1021/acs.jmedchem.3c00100 36996803

[B44] Goff B. A. Hermanto U. Rumbaugh J. Blake J. Bamberg M. Hasan T. (1994). Photoimmunotherapy and biodistribution with an OC125-chlorin immunoconjugate in an *in vivo* murine ovarian cancer model. Br. J. Cancer 70 (3), 474–480. 10.1038/bjc.1994.330 8080733 PMC2033355

[B45] Guo B. Feng Z. Hu D. Xu S. Middha E. Pan Y. (2019). Precise deciphering of brain vasculatures and microscopic tumors with dual NIR-II fluorescence and photoacoustic imaging. Adv. Mater 31 (30), e1902504. 10.1002/adma.201902504 31169334

[B46] Han X. Zhong Y. Mi C. He Z. Gu J. Dai X. (2023). NIR-IIb fluorescence-image guided synergistic surgery/starvation/chemodynamic therapy: an innovative treatment paradigm for malignant non-small cell lung cancers. Theranostics 13 (7), 2176–2191. 10.7150/thno.83753 37153731 PMC10157733

[B47] He S. Song J. Qu J. Cheng Z. (2018). Crucial breakthrough of second near-infrared biological window fluorophores: design and synthesis toward multimodal imaging and theranostics. Chem. Soc. Rev. 47 (12), 4258–4278. 10.1039/c8cs00234g 29725670

[B48] He G. Xu N. Ge H. Lu Y. Wang R. Wang H. (2021). Red-light-responsive Ru complex photosensitizer for lysosome localization photodynamic therapy. ACS Appl. Mater Interfaces 13 (17), 19572–19580. 10.1021/acsami.0c22551 33900720

[B49] Hong G. Diao S. Chang J. Antaris A. L. Chen C. Zhang B. (2014). Through-skull fluorescence imaging of the brain in a new near-infrared window. Nat. Photonics 8 (9), 723–730. 10.1038/nphoton.2014.166 27642366 PMC5026222

[B50] Hu Z. Fang C. Li B. Zhang Z. Cao C. Cai M. (2020). First-in-human liver-tumour surgery guided by multispectral fluorescence imaging in the visible and near-infrared-I/II windows. Nat. Biomed. Eng. 4 (3), 259–271. 10.1038/s41551-019-0494-0 31873212

[B51] Huang J. Pu K. (2020a). Activatable molecular probes for second near-infrared fluorescence, chemiluminescence, and photoacoustic imaging. Angew. Chem. Int. Ed. Engl. 59 (29), 11717–11731. 10.1002/anie.202001783 32134156

[B52] Huang J. Pu K. (2020b). Near-infrared fluorescent molecular probes for imaging and diagnosis of nephro-urological diseases. Chem. Sci. 12 (10), 3379–3392. 10.1039/d0sc02925d 34163613 PMC8179423

[B53] Huang Y. Guan Z. Dai X. Shen Y. Wei Q. Ren L. (2021). Engineered macrophages as near-infrared light activated drug vectors for chemo-photodynamic therapy of primary and bone metastatic breast cancer. Nat. Commun. 12 (1), 4310. 10.1038/s41467-021-24564-0 34262026 PMC8280231

[B54] Inagaki F. F. Furusawa A. Choyke P. L. Kobayashi H. (2019). Enhanced nanodrug delivery in tumors after near-infrared photoimmunotherapy. Nanophotonics 8 (10), 1673–1688. 10.1515/nanoph-2019-0186

[B55] Issanov A. Aravindakshan A. Puil L. Tammemägi M. C. Lam S. Dummer T. J. B. (2024). Risk prediction models for lung cancer in people who have never smoked: a protocol of a systematic review. Diagn Progn. Res. 8 (1), 3. 10.1186/s41512-024-00166-4 38347647 PMC10863273

[B56] Jia Q. Zhang R. Wang Y. Yan H. Li Z. Feng Y. (2022). A metabolic acidity-activatable calcium phosphate probe with fluorescence signal amplification capabilities for non-invasive imaging of tumor malignancy. Sci. Bull. (Beijing) 67 (3), 288–298. 10.1016/j.scib.2021.11.003 36546078

[B57] Kang H. Kang M. W. Kashiwagi S. Choi H. S. (2022). NIR fluorescence imaging and treatment for cancer immunotherapy. J. Immunother. Cancer 10 (7), e004936. 10.1136/jitc-2022-004936 35858710 PMC9305898

[B58] Kato T. Wakiyama H. Furusawa A. Choyke P. L. Kobayashi H. (2021). Near infrared photoimmunotherapy; A review of targets for cancer therapy. Cancers (Basel) 13 (11), 2535. 10.3390/cancers13112535 34064074 PMC8196790

[B59] Kennedy G. T. Azari F. S. Bernstein E. Nadeem B. Chang A. Segil A. (2022a). Targeted detection of cancer at the cellular level during biopsy by near-infrared confocal laser endomicroscopy. Nat. Commun. 13 (1), 2711. 10.1038/s41467-022-30265-z 35581212 PMC9114105

[B60] Kennedy G. T. Azari F. S. Bernstein E. Nadeem B. Chang A. Segil A. (2022b). Targeted detection of cancer cells during biopsy allows real-time diagnosis of pulmonary nodules. Eur. J. Nucl. Med. Mol. Imaging 49 (12), 4194–4204. 10.1007/s00259-022-05868-9 35788703 PMC9525441

[B61] Kim S. H. Park J. H. Kwon J. S. Cho J. G. Park K. G. Park C. H. (2020). NIR fluorescence for monitoring *in vivo* scaffold degradation along with stem cell tracking in bone tissue engineering. Biomaterials 258, 120267. 10.1016/j.biomaterials.2020.120267 32781325 PMC7484145

[B62] Kim S. H. Kwon J. S. Cho J. G. Park K. G. Lim T. H. Kim M. S. (2021). Non-invasive *in vivo* monitoring of transplanted stem cells in 3D-bioprinted constructs using near-infrared fluorescent imaging. Bioeng. Transl. Med. 6 (2), e10216. 10.1002/btm2.10216 34027098 PMC8126817

[B63] Kondepati V. R. Heise H. M. Backhaus J. (2008). Recent applications of near-infrared spectroscopy in cancer diagnosis and therapy. Anal. Bioanal. Chem. 390 (1), 125–139. 10.1007/s00216-007-1651-y 17955220

[B64] Lan L. Pengyuan L. Wu J. Zhibing W. (2023). Advancements in modifying the efficacy of immunotherapies through the thermal effects of nanomaterials. Nano TransMed 2 (4), 100022. 10.1016/j.ntm.2023.100022

[B65] Lauwerends L. J. van Driel P. Baatenburg de Jong R. J. Hardillo J. A. U. Koljenovic S. Puppels G. (2021). Real-time fluorescence imaging in intraoperative decision making for cancer surgery. Lancet Oncol. 22 (5), e186–e195. 10.1016/s1470-2045(20)30600-8 33765422

[B66] Li Y. Li X. Xue Z. Jiang M. Zeng S. Hao J. (2018). Second near-infrared emissive lanthanide complex for fast renal-clearable *in vivo* optical bioimaging and tiny tumor detection. Biomaterials 169, 35–44. 10.1016/j.biomaterials.2018.03.041 29631166

[B67] Li J. Jiang R. Wang Q. Li X. Hu X. Yuan Y. (2019). Semiconducting polymer nanotheranostics for NIR-II/Photoacoustic imaging-guided photothermal initiated nitric oxide/photothermal therapy. Biomaterials 217, 119304. 10.1016/j.biomaterials.2019.119304 31279099

[B68] Li X. Jiang M. Li Y. Xue Z. Zeng S. Liu H. (2019a). 808 nm laser-triggered NIR-II emissive rare-earth nanoprobes for small tumor detection and blood vessel imaging. Mater Sci. Eng. C Mater Biol. Appl. 100, 260–268. 10.1016/j.msec.2019.02.106 30948060

[B69] Li X. Wu M. Wang J. Dou Y. Gong X. Liu Y. (2019b). Ultrasmall bimodal nanomolecules enhanced tumor angiogenesis contrast with endothelial cell targeting and molecular pharmacokinetics. Nanomedicine 15 (1), 252–263. 10.1016/j.nano.2018.10.004 30359756

[B70] Li C. Chen G. Zhang Y. Wu F. Wang Q. (2020). Advanced fluorescence imaging technology in the Near-Infrared-II window for biomedical applications. J. Am. Chem. Soc. 142 (35), 14789–14804. 10.1021/jacs.0c07022 32786771

[B8] Li L. Dong X. Li J. Wei J. (2020). A short review on NIR-II organic small molecule dyes. Dyes Pigments 183, 108756. 10.1016/j.dyepig.2020.108756

[B71] Li S. Chen H. Liu H. Liu L. Yuan Y. Mao C. (2020). *In vivo* real-time pharmaceutical evaluations of near-infrared II fluorescent nanomedicine bound polyethylene glycol ligands for tumor photothermal ablation. ACS Nano 14 (10), 13681–13690. 10.1021/acsnano.0c05885 32926626

[B72] Li X. Lovell J. F. Yoon J. Chen X. (2020). Clinical development and potential of photothermal and photodynamic therapies for cancer. Nat. Rev. Clin. Oncol. 17 (11), 657–674. 10.1038/s41571-020-0410-2 32699309

[B73] Li C. H. Lv W. Y. Yan Y. Yang F. F. Zhen S. J. Huang C. Z. (2021). Nucleolin-targeted DNA nanotube for precise cancer therapy through förster resonance energy transfer-indicated telomerase responsiveness. Anal. Chem. 93 (7), 3526–3534. 10.1021/acs.analchem.0c04917 33562958

[B74] Li G. Wang Q. Liu J. Wu M. Ji H. Qin Y. (2021). Innovative strategies for enhanced tumor photodynamic therapy. J. Mater Chem. B 9 (36), 7347–7370. 10.1039/d1tb01466h 34382629

[B75] Li M. Y. Liu L. Z. Dong M. (2021). Progress on pivotal role and application of exosome in lung cancer carcinogenesis, diagnosis, therapy and prognosis. Mol. Cancer 20 (1), 22. 10.1186/s12943-021-01312-y 33504342 PMC7839206

[B76] Li X. Fang F. Sun B. Yin C. Tan J. Wan Y. (2021a). Near-infrared small molecule coupled with rigidness and flexibility for high-performance multimodal imaging-guided photodynamic and photothermal synergistic therapy. Nanoscale Horiz. 6 (2), 177–185. 10.1039/d0nh00672f 33443277

[B77] Li X. Li J. Li C. Guo Q. Wu M. Su L. (2021b). Aminopeptidase N-targeting nanomolecule-assisted delivery of VEGF siRNA to potentiate antitumour therapy by suppressing tumour revascularization and enhancing radiation response. J. Mater Chem. B 9 (36), 7530–7543. 10.1039/d1tb00990g 34551051

[B78] Li Y. Su Y. Pan H. Deng W. Wang J. Liu D. (2022). Nanodiamond-based multifunctional platform for oral chemo-photothermal combinational therapy of orthotopic Colon cancer. Pharmacol. Res. 176, 106080. 10.1016/j.phrs.2022.106080 35032663

[B79] Li C. Mi J. Wang Y. Zhang Z. Guo X. Zhou J. (2023). New and effective EGFR-Targeted fluorescence imaging technology for intraoperative rapid determination of lung cancer in freshly isolated tissue. Eur. J. Nucl. Med. Mol. Imaging 50 (2), 494–507. 10.1007/s00259-022-05975-7 36207638

[B80] Lim Y. T. Cho M. Y. Noh Y. W. Chung J. W. Chung B. H. (2009). Near-infrared emitting fluorescent nanocrystals-labeled natural killer cells as a platform technology for the optical imaging of immunotherapeutic cells-based cancer therapy. Nanotechnology 20 (47), 475102. 10.1088/0957-4484/20/47/475102 19875875

[B81] Liu Y. Fan H. Guo Q. Jiang A. Du X. Zhou J. (2017). Ultra-small pH-responsive Nd-doped NaDyF(4) nanoagents for enhanced cancer theranostic by *in situ* aggregation. Theranostics 7 (17), 4217–4228. 10.7150/thno.21557 29158821 PMC5695008

[B82] Lulevich V. Shih Y. P. Lo S. H. Liu G. Y. (2009). Cell tracing dyes significantly change single cell mechanics. J. Phys. Chem. B 113 (18), 6511–6519. 10.1021/jp8103358 19366241 PMC2698996

[B83] Mellanby R. J. Scott J. I. Mair I. Fernandez A. Saul L. Arlt J. (2018). Tricarbocyanine N-triazoles: the scaffold-of-choice for long-term near-infrared imaging of immune cells *in vivo* . Chem. Sci. 9 (36), 7261–7270. 10.1039/c8sc00900g 30288247 PMC6148684

[B84] Miaomiao X. Yuelin J. Jiajia Z. Guochen B. Xianzhu L. Le Z. (2024). NIR light-controlled DNA nanodevice for amplified mRNA imaging and precise gene therapy. Nano Today 54, 102110. 10.1016/j.nantod.2023.102110

[B85] Nagaya T. Sato K. Harada T. Nakamura Y. Choyke P. L. Kobayashi H. (2015). Near infrared photoimmunotherapy targeting EGFR positive triple negative breast cancer: optimizing the conjugate-light regimen. PLoS One 10 (8), e0136829. 10.1371/journal.pone.0136829 26313651 PMC4552472

[B86] Ogawa M. Tomita Y. Nakamura Y. Lee M. J. Lee S. Tomita S. (2017). Immunogenic cancer cell death selectively induced by near infrared photoimmunotherapy initiates host tumor immunity. Oncotarget 8 (6), 10425–10436. 10.18632/oncotarget.14425 28060726 PMC5354669

[B87] Okubo K. Takeda R. Murayama S. Umezawa M. Kamimura M. Osada K. (2021). Size-controlled bimodal *in vivo* nanoprobes as near-infrared phosphors and positive contrast agents for magnetic resonance imaging. Sci. Technol. Adv. Mater 22 (1), 160–172. 10.1080/14686996.2021.1887712 33762891 PMC7952065

[B88] Park G. K. Lee J. H. Levitz A. El Fakhri G. Hwang N. S. Henary M. (2019). Lysosome-targeted bioprobes for sequential cell tracking from macroscopic to microscopic scales. Adv. Mater 31 (14), e1806216. 10.1002/adma.201806216 30740778 PMC6574216

[B89] Ren F. Ding L. Liu H. Huang Q. Zhang H. Zhang L. (2018). Ultra-small nanocluster mediated synthesis of Nd(3+)-doped core-shell nanocrystals with emission in the second near-infrared window for multimodal imaging of tumor vasculature. Biomaterials 175, 30–43. 10.1016/j.biomaterials.2018.05.021 29800756

[B90] Ren Y. He S. Huttad L. Chua M. S. So S. K. Guo Q. (2020). An NIR-II/MR dual modal nanoprobe for liver cancer imaging. Nanoscale 12 (21), 11510–11517. 10.1039/d0nr00075b 32428058 PMC7959510

[B91] Restifo N. P. Dudley M. E. Rosenberg S. A. (2012). Adoptive immunotherapy for cancer: harnessing the T cell response. Nat. Rev. Immunol. 12 (4), 269–281. 10.1038/nri3191 22437939 PMC6292222

[B92] Sato K. Ando K. Okuyama S. Moriguchi S. Ogura T. Totoki S. (2018). Photoinduced ligand release from a silicon phthalocyanine dye conjugated with monoclonal antibodies: a mechanism of cancer cell cytotoxicity after near-infrared photoimmunotherapy. ACS Cent. Sci. 4 (11), 1559–1569. 10.1021/acscentsci.8b00565 30555909 PMC6276043

[B93] Shen L. Li J. Wen C. Wang H. Liu N. Su X. (2024). A firm-push-to-open and light-push-to-lock strategy for a general chemical platform to develop activatable dual-modality NIR-II probes. Sci. Adv. 10 (24), eado2037. 10.1126/sciadv.ado2037 38875326 PMC11177897

[B94] Shinn J. Lee S. Lee H. K. Ahn J. Lee S. A. Lee S. (2021). Recent progress in development and applications of second near-infrared (NIR-II) nanoprobes. Arch. Pharm. Res. 44 (2), 165–181. 10.1007/s12272-021-01313-x 33538959

[B95] Sies H. Belousov V. V. Chandel N. S. Davies M. J. Jones D. P. Mann G. E. (2022). Defining roles of specific reactive oxygen species (ROS) in cell biology and physiology. Nat. Rev. Mol. Cell Biol. 23 (7), 499–515. 10.1038/s41580-022-00456-z 35190722

[B96] Smith A. M. Mancini M. C. Nie S. (2009). Bioimaging: second window for *in vivo* imaging. Nat. Nanotechnol. 4 (11), 710–711. 10.1038/nnano.2009.326 19898521 PMC2862008

[B97] Su Y. Zhang Q. Miao X. Wen S. Yu S. Chu Y. (2019). Spatially engineered janus hybrid nanozyme toward SERS liquid biopsy at nano/microscales. ACS Appl. Mater Interfaces 11 (45), 41979–41987. 10.1021/acsami.9b17618 31621282

[B98] Sun Y. Qu C. Chen H. He M. Tang C. Shou K. (2016). Novel benzo-bis(1,2,5-thiadiazole) fluorophores for *in vivo* NIR-II imaging of cancer. Chem. Sci. 7 (9), 6203–6207. 10.1039/c6sc01561a 30034761 PMC6024204

[B99] Sun J. Cai W. Sun Y. Guo C. Zhang R. (2020). Facile synthesis of melanin-dye nanoagent for NIR-II fluorescence/photoacoustic imaging-guided photothermal therapy. Int. J. Nanomedicine 15, 10199–10213. 10.2147/ijn.S284520 33364754 PMC7751739

[B100] Thankarajan E. Jadhav S. Luboshits G. Gellerman G. Patsenker L. (2021). Quantification of drug release degree *in vivo* using antibody-guided, Dual-NIR-Dye ratiometric system. Anal. Chem. 93 (23), 8265–8272. 10.1021/acs.analchem.1c01104 34080851

[B101] Thanoon M. A. Zulkifley M. A. Mohd Zainuri M. A. A. Abdani S. R. (2023). A review of deep learning techniques for lung cancer screening and diagnosis based on CT images. Diagn. (Basel) 13 (16), 2617. 10.3390/diagnostics13162617 37627876 PMC10453592

[B102] Vandghanooni S. Eskandani M. Sanaat Z. Omidi Y. (2022). Recent advances in the production, reprogramming, and application of CAR-T cells for treating hematological malignancies. Life Sci. 309, 121016. 10.1016/j.lfs.2022.121016 36179813

[B103] Wan H. Du H. Wang F. Dai H. (2019). Molecular imaging in the second near-infrared window. Adv. Funct. Mater 29 (25), 1900566. 10.1002/adfm.201900566 31885529 PMC6934177

[B104] Wang S. Hüttmann G. Zhang Z. Vogel A. Birngruber R. Tangutoori S. (2015). Light-controlled delivery of monoclonal antibodies for targeted photoinactivation of Ki-67. Mol. Pharm. 12 (9), 3272–3281. 10.1021/acs.molpharmaceut.5b00260 26226545

[B105] Wang J. Wu M. Chang J. Li L. Guo Q. Hao J. (2019). Scavenger receptor-AI-targeted ultrasmall gold nanoclusters facilitate *in vivo* MR and *ex vivo* fluorescence dual-modality visualization of vulnerable atherosclerotic plaques. Nanomedicine 19, 81–94. 10.1016/j.nano.2019.04.003 31028886

[B106] Wang Z. Xia H. Chen B. Wang Y. Yin Q. Yan Y. (2021). pH-Amplified CRET nanoparticles for *in vivo* imaging of tumor metastatic lymph nodes. Angew. Chem. Int. Ed. Engl. 60 (26), 14512–14520. 10.1002/anie.202102044 33860575

[B107] Wang S. Shi H. Wang L. Loredo A. Bachilo S. M. Wu W. (2022). Photostable small-molecule NIR-II fluorescent scaffolds that cross the blood-brain barrier for noninvasive brain imaging. J. Am. Chem. Soc. 144 (51), 23668–23676. 10.1021/jacs.2c11223 36511618 PMC10010776

[B108] Wei D. Qi J. Hamblin M. R. Wen X. Jiang X. Yang H. (2022). Near-infrared photoimmunotherapy: design and potential applications for cancer treatment and beyond. Theranostics 12 (16), 7108–7131. 10.7150/thno.74820 36276636 PMC9576624

[B109] Wei H. Qi W. Liao W. Xu J. Lin C. Wang T. (2025). Multidimensional light field endoscope for robotic and AI guided surgery. Opt. Express 33 (11), 22308–22324. 10.1364/oe.559849 40515224

[B110] Welsher K. Liu Z. Sherlock S. P. Robinson J. T. Chen Z. Daranciang D. (2009). A route to brightly fluorescent carbon nanotubes for near-infrared imaging in mice. Nat. Nanotechnol. 4 (11), 773–780. 10.1038/nnano.2009.294 19893526 PMC2834239

[B111] Wu J. Liu J. Lin B. Lv R. Yuan Y. Tao X. (2021). Met-targeted dual-modal MRI/NIR II imaging for specific recognition of head and neck squamous cell carcinoma. ACS Biomater. Sci. Eng. 7 (4), 1640–1650. 10.1021/acsbiomaterials.0c01807 33719394

[B112] Wu M. Li X. Guo Q. Li J. Xu G. Li G. (2021). Magnetic mesoporous silica nanoparticles-aided dual MR/NIRF imaging to identify macrophage enrichment in atherosclerotic plaques. Nanomedicine 32, 102330. 10.1016/j.nano.2020.102330 33171287

[B113] Wu Y. Suo Y. Wang Z. Yu Y. Duan S. Liu H. (2022). First clinical applications for the NIR-II imaging with ICG in microsurgery. Front. Bioeng. Biotechnol. 10, 1042546. 10.3389/fbioe.2022.1042546 36329697 PMC9623121

[B114] Wu J. Wu G. L. Yang Q. (2024). Illuminating the future: NIR-II visualizes gas therapy for precision cancer treatment. Med. Gas. Res. 14 (4), 172–174. 10.4103/mgr.MEDGASRES-D-23-00060 40434382 PMC11257177

[B115] Wu X. Fan Y. Wang K. Miao Y. Chang Y. Ming J. (2024). NIR-II imaging-guided precise photodynamic therapy for augmenting tumor-starvation therapy by glucose metabolism reprogramming interference. Sci. Bull. (Beijing) 69 (9), 1263–1274. 10.1016/j.scib.2024.02.008 38418300

[B116] Xie J. Wang Y. Choi W. Jangili P. Ge Y. Xu Y. (2021). Overcoming barriers in photodynamic therapy harnessing nano-formulation strategies. Chem. Soc. Rev. 50 (16), 9152–9201. 10.1039/d0cs01370f 34223847

[B117] Xu Y. Ren F. Liu H. Zhang H. Han Y. Liu Z. (2019). Cholesterol-modified black phosphorus nanospheres for the first NIR-II fluorescence bioimaging. ACS Appl. Mater Interfaces 11 (24), 21399–21407. 10.1021/acsami.9b05825 31120234

[B118] Xu D. Ge J. An Y. Bai S. Wang Z. Wu S. (2023). Molecular engineering of NIR-II/IIb emitting AIEgen for multimodal imaging-guided photo-immunotherapy. Small 19 (32), e2300859. 10.1002/smll.202300859 37066745

[B119] Xu L. Fan L. Zhu J. (2023). A rare-earth near-infrared nanoprobe for the identification of small cell lung cancer. Int. J. Nanomedicine 18, 5579–5590. 10.2147/ijn.S431631 37808456 PMC10557511

[B120] Y. L. L. Z. J. L. M. Z. Y. L. D. H. (2023). 336P dual-mode near-infrared multispectral imaging system equipped with deep learning models improves the identification of cancer foci in breast cancer specimens. Ann. Oncol. 34 (S2), S317. 10.1016/j.Annonc.2023.09.532

[B121] Yang T. Tang Y. Liu L. Lv X. Wang Q. Ke H. (2017). Size-dependent Ag(2)S nanodots for second near-infrared fluorescence/photoacoustics imaging and simultaneous photothermal therapy. ACS Nano 11 (2), 1848–1857. 10.1021/acsnano.6b07866 28117993

[B122] Yang G. Mu X. Pan X. Tang Y. Yao Q. Wang Y. (2023). Ligand engineering of Au(44) nanoclusters for NIR-II luminescent and photoacoustic imaging-guided cancer photothermal therapy. Chem. Sci. 14 (16), 4308–4318. 10.1039/d2sc05729h 37123188 PMC10132122

[B123] Yu G. Durduran T. Zhou C. Wang H. W. Putt M. E. Saunders H. M. (2005). Noninvasive monitoring of murine tumor blood flow during and after photodynamic therapy provides early assessment of therapeutic efficacy. Clin. Cancer Res. 11 (9), 3543–3552. 10.1158/1078-0432.Ccr-04-2582 15867258

[B124] Zhang Y. Tao H. Li Q. Sheng W. Xu Y. Hao E. (2020). Surfactant-stripped J-aggregates of azaBODIPY derivatives: all-in-one phototheranostics in the second near infrared window. J. Control Release 326, 256–264. 10.1016/j.jconrel.2020.07.017 32682904

[B125] Zhang Z. Xu W. Kang M. Wen H. Guo H. Zhang P. (2020). An all-round athlete on the track of phototheranostics: subtly regulating the balance between radiative and nonradiative decays for multimodal imaging-guided synergistic therapy. Adv. Mater 32 (36), e2003210. 10.1002/adma.202003210 32696561

[B126] Zhang W. Zhou Y. Li D. Ma T. (2021). Near-infrared fluorescent probe with large stokes shift for detecting human neutrophil elastase in living cells. Spectrochim. Acta A Mol. Biomol. Spectrosc. 252, 119533. 10.1016/j.saa.2021.119533 33581578

[B127] Zhang X. Wang W. Su L. Ge X. Ye J. Zhao C. (2021). Plasmonic-fluorescent janus Ag/Ag(2)S nanoparticles for *in situ* H(2)O(2)-Activated NIR-II fluorescence imaging. Nano Lett. 21 (6), 2625–2633. 10.1021/acs.nanolett.1c00197 33683889

[B128] Zhang Y. Shen Q. Li Q. He P. Li J. Huang F. (2021). Ultrathin two-dimensional plasmonic PtAg nanosheets for broadband phototheranostics in both NIR-I and NIR-II biowindows. Adv. Sci. (Weinh) 8 (17), e2100386. 10.1002/advs.202100386 34247445 PMC8425935

[B129] Zhang P. Ouyang Y. Sohn Y. S. Fadeev M. Karmi O. Nechushtai R. (2022). miRNA-Guided imaging and photodynamic therapy treatment of cancer cells using Zn(II)-Protoporphyrin IX-Loaded metal-organic framework nanoparticles. ACS Nano 16 (2), 1791–1801. 10.1021/acsnano.1c04681 35020370 PMC8867907

[B130] Zhang Y. Yue X. Yang S. Li X. Cui L. Cui X. (2022). Long circulation and tumor-targeting biomimetic nanoparticles for efficient chemo/photothermal synergistic therapy. J. Mater Chem. B 10 (26), 5035–5044. 10.1039/d2tb00748g 35726686

[B131] Zhang Z. He K. Chi C. Hu Z. Tian J. (2022). Intraoperative fluorescence molecular imaging accelerates the coming of precision surgery in China. Eur. J. Nucl. Med. Mol. Imaging 49 (8), 2531–2543. 10.1007/s00259-022-05730-y 35230491 PMC9206608

[B132] Zhang Y. Jia Y. Zhu S. (2023). NIR‐II cyanine@albumin fluorophore for deep tissue imaging and imaging‐guided surgery. SmartMat 5 (4), e1245. 10.1002/smm2.1245

[B133] Zhang J. Ma W. Yang B. Shi T. Liao S. Li Y. (2024). Biomimetic metallacage nanoparticles with aggregation-induced emission for NIR-II fluorescence imaging-guided synergistic immuno-phototherapy of tumors. ACS Appl. Mater Interfaces 16 (50), 69028–69044. 10.1021/acsami.4c17413 39632260

[B134] Zhang Z. Du Y. Shi X. Wang K. Qu Q. Liang Q. (2024). NIR-II light in clinical oncology: opportunities and challenges. Nat. Reviews. Clin. Oncology 21 (6), 449–467. 10.1038/s41571-024-00892-0 38693335

[B135] Zhao D. H. Yang X. Q. Hou X. L. Xuan Y. Song X. L. Zhao Y. D. (2019). *In situ* aqueous synthesis of genetically engineered polypeptide-capped Ag(2)S quantum dots for second near-infrared fluorescence/photoacoustic imaging and photothermal therapy. J. Mater Chem. B 7 (15), 2484–2492. 10.1039/c8tb03043j 32255125

[B136] Zhao M. Li B. Zhang H. Zhang F. (2020). Activatable fluorescence sensors for *in vivo* bio-detection in the second near-infrared window. Chem. Sci. 12 (10), 3448–3459. 10.1039/d0sc04789a 34163618 PMC8179418

[B137] Zheng Z. Chen Q. Dai R. Jia Z. Yang C. Peng X. (2020a). A continuous stimuli-responsive system for NIR-II fluorescence/photoacoustic imaging guided photothermal/gas synergistic therapy. Nanoscale 12 (21), 11562–11572. 10.1039/d0nr02543g 32432283

[B138] Zheng Z. Dai R. Jia Z. Yang X. Qin Y. Rong S. (2020b). Biodegradable multifunctional nanotheranostic based on Ag(2)S-Doped hollow BSA-SiO(2) for enhancing ROS-feedback synergistic antitumor therapy. ACS Appl. Mater Interfaces 12 (49), 54356–54366. 10.1021/acsami.0c14855 33237737

[B139] Zhou C. Choe R. Shah N. Durduran T. Yu G. Durkin A. (2007). Diffuse optical monitoring of blood flow and oxygenation in human breast cancer during early stages of neoadjuvant chemotherapy. J. Biomed. Opt. 12 (5), 051903. 10.1117/1.2798595 17994886

[B140] Zhou L. Hu C. Huang H. Ge H. Zhang Z. Cheng W. (2025). Precision screening and surgical resection of pan-cancer using a tandem-locked NIR-II fluorescent probe with optimized activation efficiency. Angew. Chem. Int. Ed. Engl. 64 (36), e202509372. 10.1002/anie.202509372 40635656

